# A Pilot One and Two‐Year Prospective, Blinded Clinical Evaluation of Efficacy, and Safety of Combined Treatment With Crosslinked Hyaluronic Acid Dermal Filler and Barbed Polydioxanone Suspension Threads for Mid‐Face Contour Enhancement

**DOI:** 10.1111/jocd.16700

**Published:** 2024-12-18

**Authors:** Jovian Wan, Hyun Jin Park, Ho‐Sung Choi, Kyu‐Ho Yi

**Affiliations:** ^1^ Medical Research Inc. Wonju Korea; ^2^ Department of Anatomy Daegu Catholic University School of Medicine Daegu Korea; ^3^ PIENA Aesthetic Medical Clinic Seoul Korea; ^4^ Division in Anatomy and Developmental Biology, Department of Oral Biology Human Identification Research Institute, BK21 FOUR Project, Yonsei University College of Dentistry Seoul Korea; ^5^ Maylin Clinic (Apgujeong) Seoul Korea

**Keywords:** facial contour, hyaluronic acid, polydioxanone thread, rejuvenation, safety

## Abstract

**Introduction:**

The combination of hyaluronic acid (HA) fillers and polydioxanone (PDO) thread lifting is gaining popularity for mid‐face rejuvenation, especially among the Asian population. Despite the common use of these techniques, there is a paucity of long‐term studies assessing their combined efficacy and safety. This study aims to evaluate the efficacy and safety of combined HA filler and PDO thread treatment for mid‐face rejuvenation over a 24‐month period in a Korean population.

**Materials and Methods:**

This prospective, blinded, single‐center, open‐label trial included 11 Korean subjects, aged 29–70 years, with mid‐face volume loss graded 1–4 on the antero‐medial cheek fullness scale. Participants were treated with crosslinked HA dermal fillers and PDO threads. Assessments were conducted using the Global Aesthetic Improvement Scale (GAIS), investigator‐led clinical evaluations, and volumetric measurements using the Morpheus 3D system at baseline, 6, 12, and 24 months post‐treatment.

**Results:**

Quantitative analysis revealed a significant reduction in mid‐face width from an average baseline of 149.27–145.00 mm at 24 months (*p* < 0.00001). Similarly, lower‐face width decreased from 130.36 to 117.27 mm at 24 months (*p* < 0.00001). The GAIS scores demonstrated high levels of subject satisfaction, with 9 out of 11 patients reporting consistent satisfaction or improvement over 24 months. Minimal adverse events were reported, and no serious complications occurred.

**Discussion:**

The combination of HA fillers and PDO threads was effective in achieving and maintaining long‐term improvements in facial volume and contour. The Morpheus 3D system provided objective volumetric data, which supported the subjective improvements observed by patients and investigators. The study results highlight the benefits of ongoing neocollagenesis and tissue remodeling beyond the dissolution period of the materials.

**Conclusion:**

The combination of HA filler injections and PDO thread lifting offers a promising and minimally invasive option for long‐term mid‐face rejuvenation with high patient satisfaction and a favorable safety profile. Further studies are warranted to confirm these findings across diverse populations and compare this approach with other aesthetic treatments.

## 1 Introduction

Despite the diverse ethnic backgrounds within the Asian population, mid‐face enhancement using hyaluronic acid (HA) fillers and thread lifting remains a common pursuit, either as standalone treatments or in combination therapy. The prevalent aesthetic concerns, including wide, flat, and short facial features, necessitate the enhancement of mid‐face projection to achieve a more youthful and three‐dimensional appearance. Attaining an oval facial contour is often required to ensure a natural and well‐balanced outcome [1].

Despite the increasing popularity of these procedures, there is limited research examining the long‐term outcomes of combined HA filler and PDO thread lifting in the Asian population. This study aims to address this gap by exploring the efficacy and safety of combination therapy over a 2‐year period.

The aging process brings about cumulative changes in the skin, fat, muscle, and bone, which collectively shapes the mid‐face's appearance over time. Studies on involutional changes exhibit varying levels of scientific rigor and frequently present conflicting findings. Nevertheless, common aging‐related changes include a reduction in the maxillary and piriform angle, as well as alterations in the position of the orbital floor. Additionally, evidence suggests the inferior migration of mid‐face fat compartments during aging, contributing to the hollowing of the palpebromalar groove and the deepening of the nasojugal groove. Changes in the volume of the buccal extension of the buccal fat pad further exacerbate these effects [2, 3, 4].

Injectable HA fillers are widely utilized due to their versatility, safety, and efficacy in mid‐face rejuvenation. They are effective in restoring lost volume, enhancing facial contours, and providing essential structural support [5, 6, 7, 8]. Clinically, HA fillers are favored for their ease of administration, resistance to deformation post‐application, durability, biocompatibility, and reversibility with hyaluronidase [9].

Thread‐lifting procedures aimed at managing gravitational tissue ptosis are particularly popular in Southeast Asia. There has been a notable transition from the use of non‐absorbable threads to advanced dissolvable threads, which offer enhanced lifting effects. The selection of thread material plays a critical role in determining the outcomes of thread lifting, with absorbable polydioxanone (PDO) threads becoming increasingly prevalent due to their high tensile strength and gradual dissolution within the body [10, 11, 12].

There is a paucity of literature examining the efficacy and safety of combined HA filler and PDO thread therapy for mid‐face rejuvenation in the Asian population, particularly concerning long‐term outcomes. The primary objective of this study is to address this gap by investigating the efficacy and safety of this combination therapy over a 2‐year period.

## 2 Material and Method

### 2.1 Patients

Eleven Korean subjects, aged 29–70 years, were enrolled in the study. The mean age of the participants was 47.5 years (± 12.3). Eligible participants were non‐pregnant, non‐breastfeeding females or males aged 18 and above, with near‐symmetrical mid‐facial volume loss of grade 1–4 on the antero‐medial cheek fullness scale (0, full; 1, mildly sunken; 2, moderately sunken; 3, severely sunken; 4, very severely sunken). These participants sought volume restoration and overall rejuvenation of the mid‐face. Additionally, participants required to have the ability to understand and comply with the study requirements.

Individuals with a history of mid‐face thread lifting, cosmetic facial plastic surgery including tissue augmentation, deep peeling, or laser resurfacing within 6 months prior to the start of the study, or had received injections of HA, silicone, fat, permanent, or semipermanent fillers within the last 12 months, were ineligible for inclusion. Additionally, individuals with a history of adverse reactions or complications related to HA fillers or PDO threads were also excluded.

Informed consent was obtained from all subjects involved in the study. The study adhered to the principles outlined in the 1975 Declaration of Helsinki.

This study was a prospective, blinded, single‐center, open‐label trial, conducted over 2 years, from November 2021 to March 2024. Eleven subjects were injected with crosslinked HA dermal filler (e.p.t.q., JETEMA Co. Ltd., Korea) and received PDO threads (epiticon, JETEMA Co. Ltd., Korea) to their bilateral mid‐face. The HA filler was injected into the upper antero‐medial cheek to provide support to the retaining ligaments and create lifting tension, rather than to add volume to the entire cheek. The Morpheus 3D system was employed to evaluate volumetric changes and calculate facial width reductions in the mid‐face and lower‐face regions over the course of 24 months. For detailed information on the HA injection and thread lift procedure, please refer to Table 1.

**TABLE 1:** This table provides a summary of the hyaluronic acid filler injection and thread lift procedure conducted in the study.

Detail	e.p.t.q. S300 lidocaine	e.p.t.q. S500 lidocaine	Epiticon 18G	Epiticon 19G
Injection layer	Submuscular	Submuscular	Submuscular	Subcutaneous
Amount of injection per side of the face (range)	0.25–1.5 cc	0.5–1.5 cc	X	X
Number of threads per side of the face (range)	2–4 threads	2–4 threads	2–4 threads	2–5 threads

### 2.2 Assessment

All patients underwent clinical evaluations performed by two independent physicians who were blinded to the study details, along with photographic and Morpheus 3D (Morpheus Co. Ltd., Seong‐nam, Korea) scanning assessments, at baseline and at 6, 12, and 24 months after treatment completion. All images were captured under consistent positioning and lighting conditions in the photography room. Investigators also assessed upper face fullness using the antero‐medial cheek fullness scale at the same follow‐up intervals.

Subject satisfaction with aesthetic outcomes was self‐assessed using the Global Aesthetic Improvement Scale (GAIS) at 1, 3, 6, 12, and 24 months follow‐up (Table 2).

**TABLE 2:** Global aesthetic improvement scale (GAIS).

Score	Rating	Description
3	Very much improved	Optimal cosmetic result for the implant in this subject
2	Much improved	Marked improvement in appearance over initial condition, but not completely optimal for this subject; A touch‐up would slightly improve the result
1	Improved	Obvious improvement in appearance over initial condition, but a touch‐up or retreatment is indicated
0	No change	The appearance is essentially the same as in the original condition
−1	Worse	The appearance is worse than in the original condition

The primary efficacy outcome was the change in facial elevation after filler injection, which was determined by the differences in 3D scans between the baseline and each follow‐up. The secondary efficacy outcomes were the changes in the investigator‐led live assessment of the Merz scale score, changes in subject‐led live assessments of the GAIS from baseline to each follow‐up.

Adverse events associated with the procedures were investigated, and participants were required to report any such events during both the treatment and subsequent follow‐up appointments.

## 3 Results

### 3.1 Changes in Antero‐Medial Cheek Fullness

**FIGURE 1:**
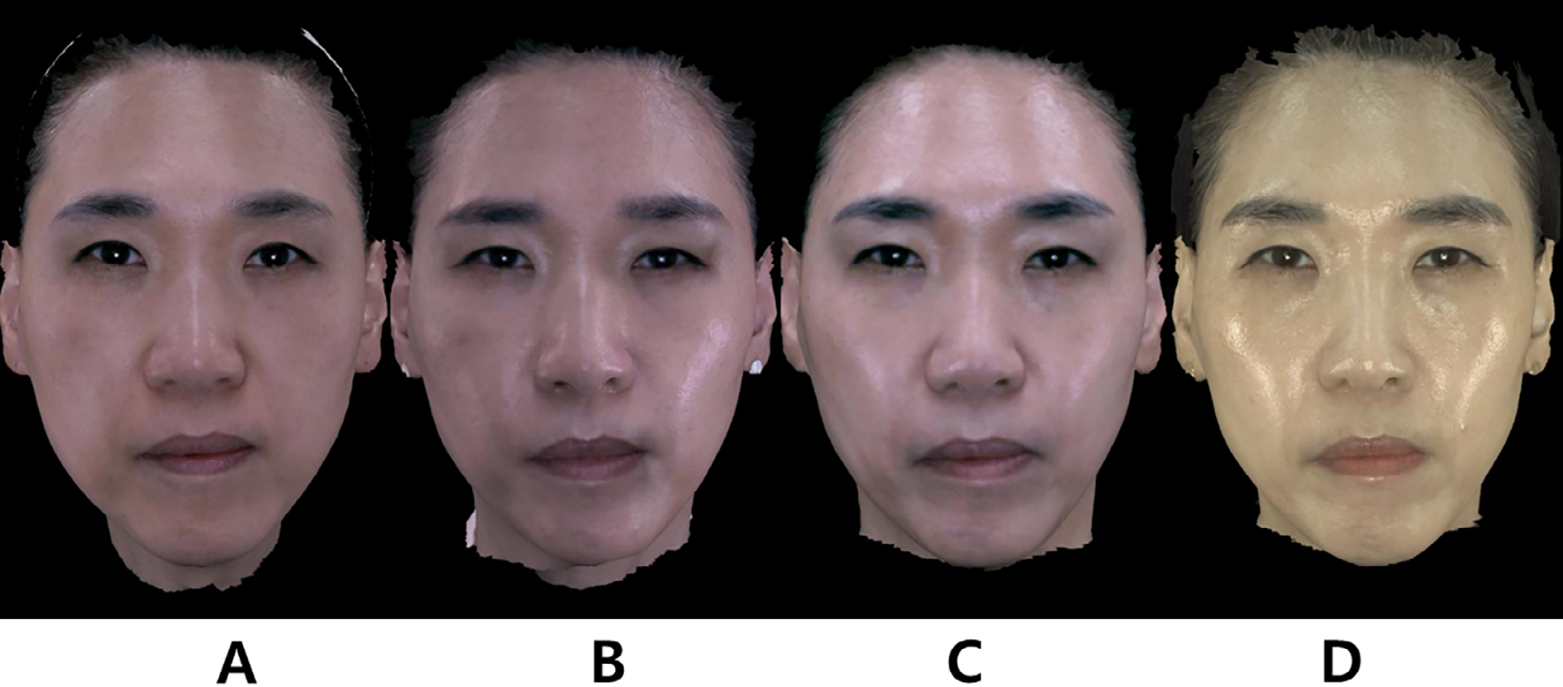
Detection of changes in mid‐face and lower‐face width after filler injection and thread lifting using the 3D scanner. Anterior views of Subject 1, a 52‐year‐old woman, showing aesthetic outcomes from combination mid‐face hyaluronic acid dermal filler and polydioxanone thread lift therapy. Three‐dimensional images of the patient are presented (A) before treatment, (B) 6 months post‐treatment, (C) 12 months post‐treatment, and (D) 24 months post‐treatment. Quantitative analysis showed a reduction in mid‐face width from 149 mm at baseline to 145 mm at 24 months, and a reduction in lower‐face width from 130 to 116 mm.

**FIGURE 2:**
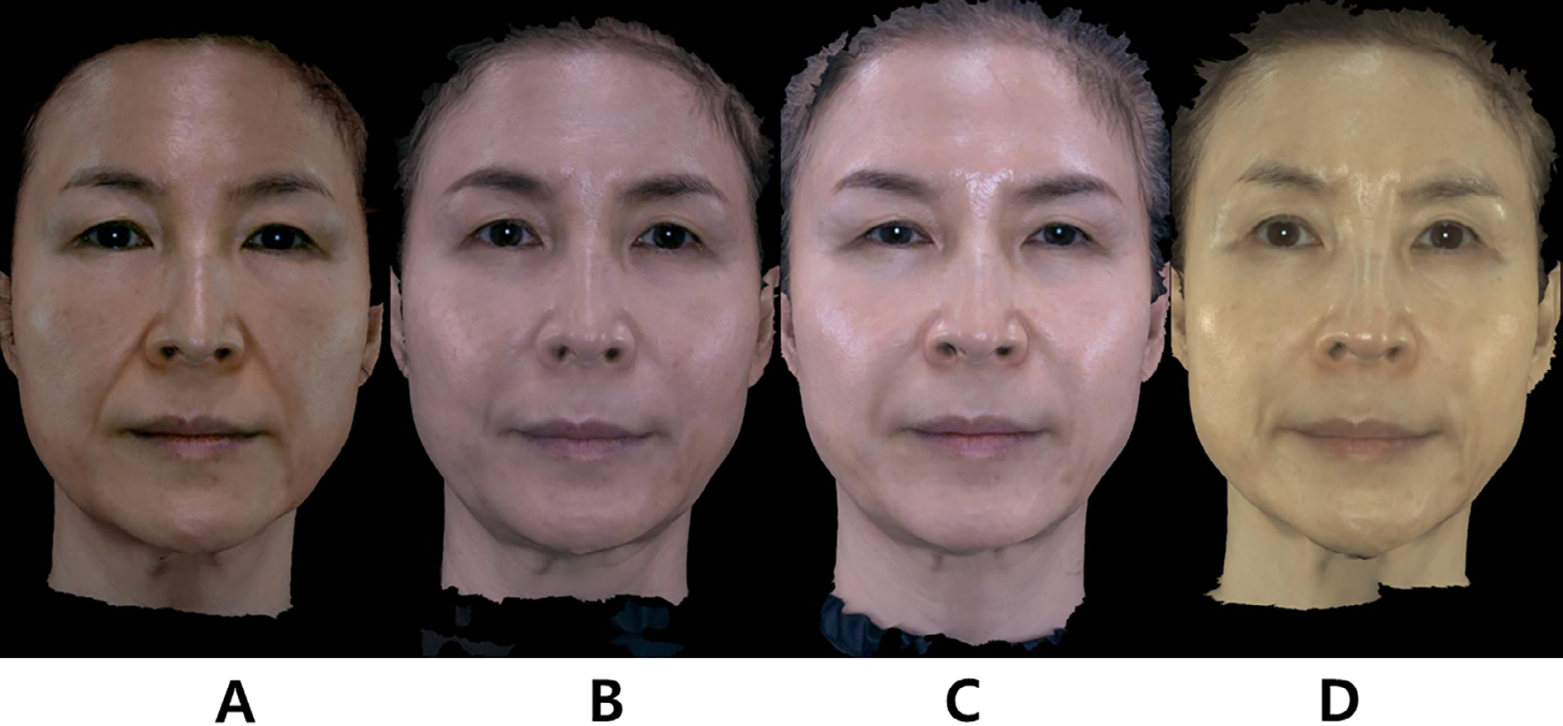
Anterior views of Subject 2, a 54‐year‐old woman, showing aesthetic outcomes from combination mid‐face hyaluronic acid dermal filler and polydioxanone thread lift therapy. Three‐dimensional images of the patient are presented (A) before treatment, (B) 6 months post‐treatment, (C) 12 months post‐treatment, and (D) 24 months post‐treatment. Volumetric changes correspond with the improvements observed in mid‐face projection.

**FIGURE 3:**
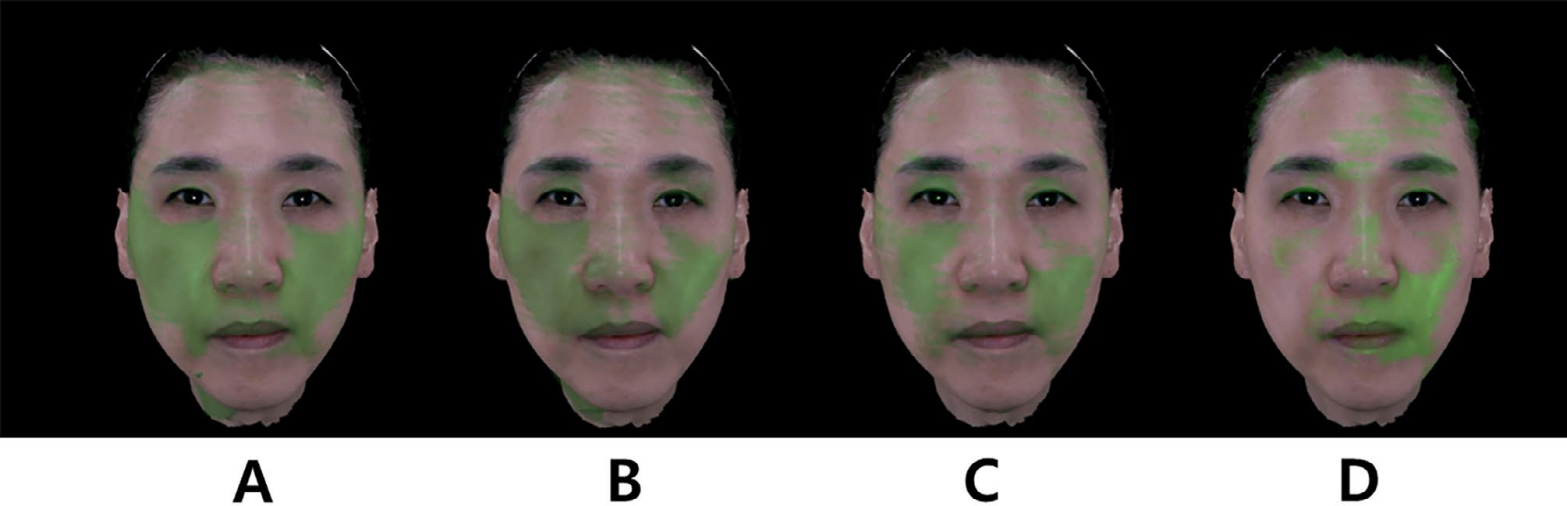
Facial volumetric changes in Subject 1, a 52‐year‐old woman, as captured by the Morpheus 3D scanner. The images demonstrate volumetric reductions in the mid‐face and lower‐face regions, with green areas indicating regions where volume has decreased. Quantitative analysis showed a reduction in mid‐face width from 149 mm at baseline to 145 mm at 24 months and a lower‐face width reduction from 130 to 116 mm over the same period.

The measures of the fullness of the antero‐medial cheek region. Here is a summary of the findings from Table 3 according to the investigators:

- *Consistent reduction in fullness*: Subjects 1, 2, 4, 5, 6, 10, and 11 showed a significant and consistent reduction in cheek fullness from baseline to 24 months. They all dropped from higher baseline scores to the lowest score of 1 by 6 months and maintained through 24 months (Figures 1, 2, 3).
- *Moderate reduction*: Subjects 3, 7, and 9 showed a reduction in cheek fullness but retained a higher score [2 or 3] over the study period. Their scores dropped initially but then stabilized, showing moderate but not complete reduction.
- *Minimal reduction*: Subject 8 showed the least amount of fullness from the start, reducing to 0 at 6 months and maintaining this score through 24 months. In this study, a score of “0” indicates “no change in fullness.”

**TABLE 3:** Patient demographics, cheek fullness, facial width changes, and GAIS scores.

Subject	Sex	Age (years)	Cheek fullness (investigator 1: baseline, 6, 12, 24 months)	Cheek fullness (investigator 2: baseline, 6, 12, 24 months)	Mid‐face width (baseline, 24 months, reduction)	Lower‐face width (baseline, 24 months, reduction)	GAIS scores (1, 3, 6, 12, 24 months)
1	Female	52	2, 1, 1, 1	3, 1, 1, 1	149, 145, 4 mm	130, 116, 14 mm	3, 2, 2, 2, 2
2	Female	54	3, 1, 1, 1	3, 1, 1, 1	150, 146, 4 mm	132, 118, 14 mm	2, 1, 1, 1, 1
3	Female	46	3, 2, 2, 2	3, 2, 2, 2	151, 147, 4 mm	133, 119, 14 mm	2, 2, 1, 1, 1
4	Female	41	2, 1, 1, 1	2, 1, 1, 1	152, 148, 4 mm	134, 120, 14 mm	2, 1, 1, 1, 1
5	Female	47	2, 1, 1, 1	2, 1, 1, 1	148, 144, 4 mm	131, 117, 14 mm	2, 2, 2, 1, 1
6	Female	50	2, 1, 1, 1	2, 1, 1, 1	147, 143, 4 mm	129, 115, 14 mm	1, 1, 1, 1, 1
7	Male	62	4, 2, 2, 2	4, 2, 2, 2	153, 149, 4 mm	135, 121, 14 mm	3, 3, 3, 3, 2
8	Female	29	1, 0, 0, 0	1, 0, 0, 0	150, 146, 4 mm	133, 118, 15 mm	2, 2, 2, 1, 1
9	Female	59	4, 3, 3, 3	4, 3, 3, 3	151, 147, 4 mm	132, 119, 13 mm	3, 2, 2, 2, 1
10	Female	44	2, 1, 1, 1	2, 1, 1, 1	149, 144, 5 mm	130, 116, 14 mm	1, 1, 1, 1, 1
11	Male	38	3, 1, 1, 1	3, 1, 1, 1	148, 143, 5 mm	131, 117, 14 mm	2, 2, 2, 2, 1

Both investigators' scores showed consistent results across subjects, indicating reliability in the observed changes.

### 3.2 Quantitative Analysis of Facial Width Reduction

The Morpheus 3D system was used to assess facial width changes in the mid‐face and lower‐face regions over 24 months. Quantitative analysis demonstrated a significant reduction in facial width across all patients, corroborating the subjective improvements observed.

- Mid‐face width: Mid‐face width decreased from an average baseline of 149.27 to 145.00 mm at 24 months (p < 0.00001), showing a consistent reduction in facial volume that aligned with investigator and patient assessments of improved mid‐face projection.
- *Lower‐face width*: ssLower‐face width also exhibited a significant reduction, decreasing from an average baseline of 130.36 to 117.27 mm at 24 months (p < 0.00001), further enhancing the definition of the lower facial contours (Table 3).

### 3.3 Global Aesthetic Improvement Scale

This GAIS evaluated subjects' perceived aesthetic improvements over time. The findings from Table 3 can be summarized as follows:

- *Consistent satisfaction*: Subjects 1, 2, 4, 6, 8, 10, and 11 reported a consistent level of satisfaction or improvement that remained stable from 1 to 24 months, indicating long‐term contentment with the results.
- *Initial improvement followed by plateau*: Subjects 3, 5, and 9 reported initial improvement, with higher scores at 1 month, which then plateaued at a lower level, suggesting that initial improvements were maintained over time without further change.
- *Sustained higher satisfaction*: Subjects 7 reported high satisfaction throughout, with scores of 3 or 2 across all time points, indicating a high level of sustained aesthetic improvement.

These findings support the long‐term efficacy and stability of the combined HA filler and PDO thread treatment for mid‐face rejuvenation.

## 4 Discussion

The aesthetic management of mid‐face aging presents significant complexity, requiring the application of diverse treatment methodologies in clinical practice. Minimally invasive approaches, such as HA fillers and PDO threads, are frequently employed, but other options, including autologous fat grafting and surgical interventions, are also well‐documented in the literature [13, 14, 15, 16, 17, 18].

In this study, the Morpheus 3D system was utilized for clinical assessment, employing structured light scanning to facilitate 3D photogrammetry imaging. Previous studies have validated the accuracy and reproducibility of results obtained with this system [19]. The findings of this study demonstrated a significant improvement in mid‐face volume, with a noticeable repositioning that contributed to enhanced upper cheek fullness. Improvements in overall facial contours were observed in all subjects, resulting in a lifted appearance and a more oval facial shape with sharper definition. These positive outcomes were evident as early as 6 months post‐treatment and persisted at the 24‐month follow‐up for all participants.

While extremely rare, serious complications related to HA filler injections have been documented, including skin necrosis, blindness, and stroke due to vascular compromise [20, 21]. The high‐risk facial areas for such complications include the glabella, nasal ala, and nasal dorsum [22, 23]. The literature emphasizes several precautions to minimize the risk of vascular complications, including a thorough understanding of facial anatomy, administering low‐pressure injections with minimal volumes, diluting the filler with lidocaine and/or epinephrine, keeping the needle in motion, avoiding injections in areas with previous scarring, and using blunt cannulas to reduce the likelihood of intravascular filler placement [24, 25, 26, 27, 28].

PDO threads are widely used in aesthetic medicine for facial rejuvenation and body contouring. Although complications such as bruising, bleeding, and redness are generally minor and transient, they are still noteworthy [29, 30]. Yi and Park [31] explored the use of PDO threads for mid‐face volumization, concluding that the tensile strength, dissolution period, and elastic modulus of PDO threads contribute to their effectiveness in tissue lifting and volumization. While Yi and Park utilized PDO cog volumizing threads, our study employed barbed PDO threads. However, research has consistently shown that the formation of a collagen tube induced by PDO threads stimulates collagen production and supports tissue, resulting in a lifting effect [32].

Yi and Park [31] also discussed potential complications of PDO thread lifting in the mid‐face region, such as thread protrusion, which can occur in the oral cavity or nasal area. In cases of protrusion, gently pulling on the exposed thread end can facilitate its release and removal. For threads that are nearly protruding, creating a small incision with a needle and carefully extracting the thread can resolve the issue. If a thread is inserted too superficially, it may contact the dermis, potentially affecting its shape. While superficially inserted threads generally improve over time, there is a risk of them developing into persistent scar tissue. Options for remediation include removing the thread through a minor incision or performing subcision and injecting substances like HA to dissolve the thread.

According to Hong et al. [12], the longevity of thread‐lifting procedures is influenced by several factors, most notably the material of the threads, with PDO threads emerging as the most commonly used. PDO threads, known for their tensile strength, take more than 6 months to dissolve completely, maintaining the lifting effect for an extended period [33, 34]. Other factors influencing the longevity of thread lifting include thread thickness, tensile strength, the shape and number of cogs, insertion depth, selection of appropriate lifting vectors and fixation points, and the expiration date of absorbable threads [12, 35, 36].

The extended follow‐up period in this study, spanning 24 months, was designed to capture not only the immediate effects of HA fillers and PDO threads but also the long‐term outcomes potentially resulting from the biological responses they induce. Although PDO threads generally dissolve within 6 months and HA fillers between 12 and 16 months, these materials can stimulate collagen production and tissue remodeling, which may lead to sustained aesthetic improvements [36]. The prolonged results observed in this study are likely due to ongoing neocollagenesis and tissue support [11, 36, 37].

There are several limitations to this study that warrant further investigation. The primary limitation is the small sample size, consisting of only 11 patients, which may restrict the statistical power and generalizability of the findings. On the other hand, a positive aspect of the study is the extended follow‐up period, spanning 24 months, which allowed for the evaluation of both short‐ and long‐term outcomes. Additionally, the subject cohort consisted entirely of Korean individuals, predominantly female, with only one male participant. Given the growing interest in aesthetic treatments among males, future studies should verify the applicability of these findings to this demographic [38]. Focusing on Korean participants with Fitzpatrick skin type III may also limit the generalizability of the results to populations with different skin types and ethnic backgrounds, as research suggests skin characteristics vary across ethnicities [39]. Another limitation is the lack of a standardized volume of HA filler across patients. Moreover, all treatments were performed by the same practitioner, which may introduce variation due to differences in technique and experience.

Finally, it is essential to consider the effects of intrinsic aging over the 2‐year study period. Despite the treatments administered, natural aging processes continued to affect patients' faces, potentially influencing the outcomes. Future studies should consider conducting randomized controlled trials comparing the effectiveness of this treatment approach with other popular aesthetic rejuvenation procedures, such as fat transfer or combination treatments involving poly‐L‐lactic acid with thread lifting.

## 5 Conclusion

In conclusion, the combination of HA filler injections and PDO thread lifting provides satisfactory outcomes for mid‐face rejuvenation over a 2‐year period. While there may be minimal potential side effects, such as instances of thread protrusion or contact, these are typically resolved through gentle removal.

## Disclosure

This study was conducted in compliance with the principles set forth in the Declaration of Helsinki.

## Conflicts of Interest

The authors declare no conflicts of interest.

## Data Availability

The data that support the findings of this study are available from the corresponding author upon reasonable request.
