# Correction to “A Pilot One and Two‐Year Prospective, Blinded Clinical Evaluation of Efficacy, and Safety of Combined Treatment With Crosslinked Hyaluronic Acid Dermal Filler and Barbed Polydioxanone Suspension Threads for Mid‐Face Contour Enhancement”

**DOI:** 10.1111/jocd.70506

**Published:** 2025-10-18

Wan J., Park H. J., Choi H.‐S., and Yi K.‐H . A pilot one‐ and two‐year prospective, blinded clinical evaluation of efficacy, and safety of combined treatment with crosslinked hyaluronic acid dermal filler and barbed polydioxanone suspension threads for mid‐face contour enhancement. J Cosmet Dermatol. 2025;24(2):e16700.39692727 10.1111/jocd.16700PMC11845956

On page 6 of 7, in the “Conflicts of Interest” section, the statement “The authors declare no conflicts of interest.” was incorrect. It should have read: “Conflicts of Interest: All authors have served as clinical advisors to JETEMA Co., Ltd. (Korea), the manufacturer of e.p.t.q. fillers and the provider of epiticon threads.”

We apologize for this error.

